# Engineered microneedle arrays for transcutaneous HIV vaccine delivery

**DOI:** 10.1186/1742-4690-9-S2-P334

**Published:** 2012-09-13

**Authors:** P DeMuth, B Huang, Y Min, D Barouch, P Hammond, D Irvine

**Affiliations:** 1 MIT, Cambridge, MA, USA; 2 Harvard Medical School, Boston, MA, USA

## Background

Needle-free delivery has the potential to enhance vaccine efficacy and safety, as well as facilitate cost-effective global vaccine distribution and storage. To this end, microneedle arrays provide the ability for pain-free, safe, and convenient materials delivery through disruption of the outer layers of the skin to access potent immune-competent cell populations residing within the epidermal/dermal tissues.

## Methods

We have employed this strategy to design a series of vaccine-delivery strategies based on the rapid delivery of dried vaccine-containing surface-coatings from biodegradable microneedles upon gentle application to the skin. We have demonstrated the utility of this approach for the rapid and convenient delivery of plasmid DNA as well as recombinant adenoviral vectors expressing model HIV antigens.

## Results

When used to deliver a plasmid expressing SIV-Gag together with poly(I:C) in mice, these engineered microneedles elicited stronger cellular and humoral immunity than traditional naked DNA injections and were comparable in potency to DNA administered with in vivo electroporation. Furthermore, microneedle delivery of a luciferase-expressing plasmid in explanted skin from Rhesus macaques showed significantly enhanced expression (~100-fold greater) relative to injected dose-matched controls. In parallel, we designed coatings to encapsulate replication-deficient adenoviral vectors, and dried microneedle-formulated Ad vaccines were observed to maintain biological activity at room temperature for in excess of 8 weeks, while control formulations rapidly lost activity within 7 days. Microneedle-delivery of adenoviral vectors expressing SIV-Gag was observed to produce cellular and humoral immune responses comparable to traditional intramuscular/intradermal injection. Thus, this microneedle-based delivery strategy has the potential to provide enhanced vaccine stability while maintaining efficacy equivalent to traditional approaches.

## Conclusion

Together, these results demonstrate the promise of microneedles for effective and safe vaccine delivery while showcasing the additional potential for greater stability in storage and more potent immunogenicity.

Supported by: Ragon Inst. of MGH, MIT, and Harvard, NIH (AI095109), Dept. of Defense (W911NF-07-D-0004).

